# COVID-19 and Global Distributive Justice: ‘Health Diplomacy’ of India and South Africa for the TRIPS waiver

**DOI:** 10.1177/00219096211069652

**Published:** 2022-01-18

**Authors:** Bawa Singh, Vijay Kumar Chattu, Jaspal Kaur, Rajni Mol, Priya Gauttam, Balinder Singh

**Affiliations:** Department of South and Central Asian Studies, School of International Studies, Central University of Punjab, India; Department of Medicine, Temerty Faculty of Medicine, University of Toronto, Canada; Department of Community Medicine, Faculty of Medicine, Datta Meghe Institute of Medical Sciences, India; Center for Transdisciplinary Research, Saveetha Institute of Medical and Technical Sciences, Saveetha University, India; Guru Nanak Dev University, India; Department of South and Central Asian Studies, School of International Studies, Central University of Punjab, India; Department of Political Science, Central University of Himachal Pradesh, India

**Keywords:** Pandemic, global solidarity, global health diplomacy, patent diplomacy, TRIPS, Intellectual Property Rights, India, World Trade Organization, South Africa

## Abstract

The second wave of the COVID-19 pandemic had left heart-wrenching impacts on all facets of life in general and the availability, accessibility, and affordability of medicines and vaccines in particular. Rather, the world has been divided into two groups regarding access to medicine and vaccines as haves and have-nots. The rich countries had pre-ordered the vaccines of COVID-19 along with the holding of the same. The pandemic situation was further worsened, given the Trade-Related Intellectual Property Rights (TRIPS) in practice and restrictions on sharing technology of vaccines, medicines, and life-saving equipment. In this context, India and South Africa have proposed the joint proposal and garnered support for waiving off TRIPS to ensure equity, accessibility, and affordability of vaccines and the same as public goods. In this review, we emphasize that global justice is one of the important elements of normative international theories, which focus on all the moral obligations from the world’s rich to the world’s poor. The paper also questions and argues that if the rich countries fail to go by the principles of global justice, can the Indian and South African (SA) patent diplomacy play a catalyst role in global justice? The review concludes with an emphasis on global solidarity, and the acceptance of joint India–South Africa’s “patent diplomacy” for TRIPS waiver would result in mass production and fair distribution, making the COVID-19 medicines and technologies available to everyone regardless of their poor–rich status.

## Introduction

There is a strong logic to connect the relationship between the pandemic and waiving off the Trade-Related Intellectual Property Rights (TRIPS) given the “access” as one of the major barriers to healthcare equity. In the post-first wave, the second wave has been on the peak, which is more deadly with some rare diseases like several variants of fungus. It is taking a heavy toll of deaths in general and particularly recording more than 400,000 daily (which has come down gradually after a month or so) cases and more than 4000 deaths in India. It has not only exposed the health infrastructures of the developing countries but rather also of the developed countries as well. Infectious diseases have been causing havoc in developed and developing societies throughout recorded history. Infectious diseases have repeatedly been occurring at unprecedented levels. The World Health Organization (WHO) has witnessed the emergence of several epidemics and pandemics caused by more than 20 infectious agents over the recent past (Suneja, 2021).

The second wave of COVID-19 had left heart-wrenching impacts on all facets of life in general and the availability, accessibility, and affordability of medicines and vaccines in particular. Given the prevailing scenario of COVID-19, the health facilities have not been equitable. Rather, the world has been divided into two groups regarding access to medicines and vaccines as haves and have-nots. The rich countries had pre-ordered the vaccines of COVID-19 along with the holding of the same. In contrast, however, the developing countries have not been able to provide vaccines to their citizens even the vaccination drive is covered the majority of the population in the developed countries. These inequities and gaps have necessitated requesting a TRIPS waiver to ensure everyone’s access, affordability, and availability of vaccines and improve healthcare equity.

In this context, it is essential to understand the inequity of healthcare facilities, particularly during epidemics or pandemics times. The pandemic situation was further worsened, given the TRIPS in practice and restrictions on sharing technology of vaccines, medicines, and life-saving equipment. Realizing the critical situation prevailing against such backgrounds of the first and second wave of COVID-19 and the division of the world into haves and not haves of medicines and vaccines, India and South Africa have proposed the joint proposal and garnering support for waiving off the TRIPS to ensure equity, accessibility, and affordability of vaccines and same as public goods. A considerable number of countries, civil societies, philanthropic societies, human rights activists, the WHO, United Nations Conference on Trade and Development (UNCTAD; WHO, n.d.-a), and many other regional and international organizations had come forward to extend their support to India and South Africa’s joint proposal of temporary waiving off the TRIPS. However, the rich countries, particularly European Union countries and Canada, Australia, New Zealand, and the United States, had staunchly opposed the Indian and African joint proposal to waive the TRIPS.

Inadequate manufacturing of vaccines and medicines, and consumer goods, particularly during the peak of a pandemic caused by the COVID-19 pandemic, is a critical issue (Valinsky, 2020). Being the “Black Swan” event, COVID-19 left indelible imprints on socio-economic facets such as declining economic growth rate, exponentially growing unemployment rate, managing and arranging the basic life necessities. Inadequate health services have made the COVID-19 pandemic one of the world’s major critical issues, even rather characterized as a black swan event. Furthermore, there is a shortage of doctors (Tiefenthäler, 2020), life-saving equipment, personal protective equipment (e.g. face masks, gloves, face shields, and sanitizing products), as well as ventilators, hospital beds, intensive care unit (ICU) beds, oxygen, and Extracorporeal Membrane Oxygenation (ECMO) machines (Valinsky, 2020). Against this background, it makes sense and moral obligation to ensure global justice in vaccines or medicines distribution for all, which is not happening now. Besides, vaccine and medicine shortages, inaccessibility, affordability and equity have been serious blots on global and distributive justice. The shortage of health infrastructure, medicine, vaccines drugs has created an important question for global governance and policymakers. An important and moot question emerges in this background: what is global justice? Is the global justice still prevailing at the moment? In this context, scholars (Wordometer, n.d.) have argued that global justice is one of the important elements of normative international theories, which focused on all the moral obligations on the part of the world’s rich to the world’s poor. As per this theoretical argument, global justice stands for redistributing wealth to poor people to eliminate global poverty and other differences. Therefore, does the simple meaning of justice resonate the same as equity and equality? In this context, this review is aimed to analyze if the Indian and SA patent diplomacy plays a catalyst role when the affluent nations fail to adhere to the principles of global justice?

## Methodology

We have conducted an extensive literature search in all the major databases such as Web of Science, Scopus, ProQuest, Embase, JSTOR, and Google scholar search engine. The search terms included “India and South Africa’s joint proposal,” “TRIPS waiver,” “COVID-19 vaccine coverage,” “Global solidarity,” and “Equitable distribution of COVID-19 vaccines.” The article titles were screened, duplicates were removed, and all the relevant published articles were included in this review. To support the argument and to find more evidence, a grey literature search from some authentic websites and reports of government, World Trade Organization (WTO), and WHO was done and included in the literature search. The major findings supporting the study objectives were gathered and classified into major subheadings, then discussed in depth in the “Results” section.

## Results

### Global COVID-19 pandemic

The Severe Acute Respiratory Syndrome Coronavirus-2 (SARS-CoV-2) identified in late December 2019 in Wuhan (China) resulted in this ongoing global COVID-19 Pandemic. Because of its fast spread across the world in a short period, the WHO declared it a Public Health Emergency of International Concern (PHEIC) on 30 January 2020 and a Pandemic on 11 March 2020, respectively. The world has been passing through multiple waves of the pandemic, wreaking havoc on health and humanity across the globe. This argument can be corroborated and substantiated by the total infected cases reaching 219,284,577, deaths 4,545,466, and recovered cases around 196,075,470 (Editorial, 2020). Besides its geographical spread, it has resulted in the worst kind of economic recession, shortage of health facilities such as doctors and paramedical staff, ICU beds, vaccines, medicines, oxygen supply, and life safety equipment (Nueangnong et al., 2020). As of 2 September 2021, more than 200 million cases have been confirmed globally, along with more than 4.51 million deaths attributed to COVID-19 (see Table 1). Because of these dreadful consequences, this epidemic has become one of the deadliest in history (Callard, 2020).

**Table 1.** Total cases of the COVID-19—global scenario (as of 16 Septembers 2021).

Country, other	Total cases	Total deaths	Total recovered	Active cases	Serious, critical	Population
The United States	42,479,780	685,023	32,271,084	9,523,673	25,461	333,267,823
India	33,347,325	443,960	32,560,474	342,891	8944	1,395,827,756
Brazil	21,034,610	588,640	20,138,267	307,703	8318	214,322,393
Russia	7,194,926	195,041	6,435,072	564,813	7,194,926	146,007,547
The United Kingdom	7,312,683	134,647	5,878,889	1,299,147	1060	68,302,217
South Africa	2,787,203	82,496	2,560,605	144,102	546	60,180,348

*Source*: Worldometer. Accessed on 16 September 2021.

Retrieved from: https://www.worldometers.info/coronavirus/.

While the initial wave of the pandemic is still ongoing in some nations, the second and third waves of the epidemic have overburdened the global healthcare system with more fatalities and disabilities. The first wave resulted in massive death tolls and lasting impacts of a SARS-2 infection, whereas even for some mild COVID-19 patients can be debilitating for months despite being clinically cured of the infection (Major and Machin, 2020). In some of the countries, the third wave has been ongoing. The third wave is considered the effects of the virus on social determinants such as health and its dreaded impacts on the next generations (Banks et al., 2020). Some scholars (Fisayo and Tsukagoshi, 2021) have argued that the virus will inflate health inequalities through severe economic implications. Concomitantly, it has also been highlighted by some scholars that low-paid staff such as women, young people, Black people, Asian, and Minority Ethnic (BAME) would take the longest time to recover from the predicted deep economic recession. The implications of health caused by the first, second, and third waves of the pandemic are likely to worsen bad economic conditions. However, the BAME groups are likely to remain at the intersection between poverty and poor health, which would suffer the most. Given this context, some scholars (Beaumont, 2020) have argued that the ongoing pandemic will not be “over” until it has been addressed in every aspect of our lives.

At the beginning of the pandemic, there was no treatment available, and to control the spread, some preventive measures such as lockdowns, social distancing, quarantine, and the banning of internal and external travels have been put in place. Beaumont (18 November 2020; EMA, n.d.) has argued that several countries had implemented phased distribution plans with the availability of vaccines. In such plans, the elderly and other people with a high risk of exposure and transmission, such as healthcare workers, were prioritized. The European Union had approved the Pfizer BioNTech vaccine, and the vaccinations were administered on 27 December 2020. The Moderna vaccine was authorized to be administered on 6 January 2021 (Kumar, 2020).

### The COVID-19 pandemic scenario in India

The global coronavirus cases have been growing exponentially since the initial epidemic, and India is no exception. The first case of coronavirus was reported on 30 January 2020. By 3 February 2020, the same cases rose to three; the patients were the students who had just returned from Wuhan (Perappadan, 2020). Perappadan (2020; Ministry of Health and Welfare Government of India, n.d.) has argued that there was no considerable growth in case transmissions till the end of February 2020. Subsequently, cases were started growing day by day, and by 9 June 2020, the total number of COVID-19 cases reached 266,598 from the 32 states/union territories (Biswas, 2020). By October 2020, the Panel on COVID-19 (GOI) stated that the ongoing pandemic had peaked (Agrawal et al., 2021). The panel’s prediction was based on the mathematical simulation (Indian Supermodel), assuming India might have reached herd immunity (Agrawal et al., 2021). On 16 January 2021, India had initiated its vaccination program (BBC News, 2021). As of 16 September 2021, about 620 million people have received at least one dose of a COVID vaccine so far. By reaching February 2021, the daily cases in India had fallen to 9000 per day (The Hindu, 2021). Being complacent in COVID-19 treatment and healthcare equity, the Government and India’s citizens have begun undoing the COVID behaviors. The second wave with mutations of B.1.617 (Delta variant) started expanding its ugly head in Maharashtra (India) by October 2020. Still, there was no seriousness on the part of the administration to look into the same. By 9 April 2021, a major second wave of infections became very critical. With more than 300,000–400,000 cases, India has started recording the world’s highest single-day spike on 21 April 2021 (Business Today, 2021). On 6 May 2021, the highest number of COVID-19 cases and deaths, that is, 412,262 and 3980 respectively, has been reported to take the country’s tally to 24,046,809 (Table 2), the latest update by the Union Health Ministry (South African Ministry of Health, 2020).

**Table 2.** COVID-19 cases in various Indian states (as of 16 September 2021).

State/UTs	Total cases	Active cases	Discharged	Deaths
Maharashtra	6,507,930	52,583,637	6,317,070	138,277
Kerala	4,424,046	191,313	4,209,746	22,987,208
Karnataka	2,964,083	15,920,138	2,910,626	375,378
Tamil Nadu	2,638,668	1,663,687	2,586,786	3,524,629
Andhra Pradesh	2,033,419	14,603,191	2,004,786	1,403,011
Uttar Pradesh	1,709,605	1821	1,686,538	228,851
West Bengal	1,558,860	805,024	1,532,197	1,861,314
Delhi	1,438,345	4044	1,412,858	25,083
Odisha	1,017,718	5,440,110	1,004,164	81,146
Chhattisgarh	1,004,957	35,021	991,048	13,559
Rajasthan	954,226	10,411	945,168	8954
Gujarat	825,655	1494	815,424	10,082
Madhya Pradesh	792,367	1242	781,726	10,517
Haryana	770,688	33,513	760,545	98,081
Bihar	725,852	668	716,128	9658
Telangana	662,526	532,543	653,302	38,991
Punjab	601,150	3256	584,361	16,464
Assam	596,606	5,396,270	585,435	57,758
Jharkhand	348,096	1101	342,853	5133
Uttarakhand	343,310	29,611	335,625	7389
Jammu and Kashmir	327,296	134,924	321,532	44,151
Himachal Pradesh	216,303	1,650,118	211,009	36,442
Goa	175,088	70,017	171,099	328,970
Puducherry	125,063	90,042	122,338	18,252
Manipur	117,697	26,217	113,259	18,172
Tripura	8,375,641	45,314	8,249,727	806
Meghalaya	78,729,243	166,425	75,698,262	13,676
Mizoram	754,701,402	13,973,448	61,247,950	2504
Chandigarh	651,642	291	643,171	818
Arunachal Pradesh	5,394,371	54,517	5,312,754	271
Sikkim	3,073,842	74,730	2,961,271	3791
Nagaland	3,073,137	51,952	2,956,689	646
Ladakh	206,259	404	203,785	207
Dadra And Nagar Haveli And Daman And Diu	10,670	5	10,661	4
Lakshadweep	10,353	4	10,298	51
Andaman And Nicobar	7592	132	74,502	129

UTs: union territories.

*Source*: https://www.mygov.in/covid-19 (accessed 16 September 2021).

### South-African pandemic scenario

Diaspora and damage due to pandemics have remained interwoven throughout recorded history. South Africa is also one of the countries with a considerable number of the diaspora in several countries. No country has remained safe globally given the internal and external milieus. South Africa is no exception to the same. The first case of a patient with COVID-19 was reported in South Africa who had returned with his wife and eight other members from Milan, Italy. The Minister of Health Zweli Mkhize had declared the first confirmed case on 5 March 2020. Following that, the National Institute for Communicable Diseases (NICD) epidemiologists and clinicians in Kwazulu-Natal were tasked, and the patient was admitted to Grey’s Hospital in Pietermaritzburg (Hoffman, 1981). According to the Worldometer’s report (as of 16 September 2021), South Africa has reported a total of 2,869,201 COVID-19 cases; 85,469 deaths; 2,700,299 recoveries; and 83,433 active COVID-19 cases. However, India has reported 33,347,325—total COVID-19 cases, 443,960 deaths, 32,560,474 total recoveries, and 342,891 active cases.

### Pandemic and global justice

With the pandemic, there is a critical shortage of vaccines. However, it is a man-made phenomenon rather than a natural one, making global justice contentious. Understanding global justice can be primarily through three approaches: Cosmopolitanism, Communitarianism, and Neorealism. The first approach, like Cosmopolitanism, used to see global justice from the individual perspective where individuals used to be considered part of the global society; meanwhile, communitarianism and neorealism are perceived as a state-centric view of justice. As far as Cosmopolitanism is concerned, this approach generally tasked the individuals with the responsibility to act as global citizens. Slaughter (2008) has grounded cosmopolitanism in two criteria invoking a commitment on the part of the individual to a universal community on one hand and detaching from the local or national affiliations on the other hand as well (Pogge, 2001). He has broken cosmopolitanism into three sub-theories-morals, institutional and political.

Communitarianism stands for a diametrically opposite strategy to cosmopolitanism, emphasizing an individual’s role within their political community. Hoffman (1991) has recommended that global justice ought to remain every state’s microcosm (McGrew, 2004). However, this approach has failed to ensure global justice in countries wherein serious inequality haunts the community. It is believed that notwithstanding the mandate of the countries to work for reducing or resolving the inequalities and injustice concerns, it would infringe on the very sovereignty of the state. Against this background, it has become worth mentioning Pogge (2001: 15), who argued that

The idea that our economic policies and global economic institutions we impose make us causally and morally responsible for the perpetuation—even aggravation—of world hunger . . . is an idea rarely taken seriously . . . in the developed world. (Vaidyanathan, 2021)

Third, neorealism and the concept of global justice have been moving in diametrically opposite directions. Given the core principles of neorealism based on the primacy of the state, state sovereignty, and security, there is no scope or place for motivation for magnanimity. Realist thinkers (WHO, n.d.-b) had argued that systemic economic inequalities are unavoidable in international relations (IR; McGrew, 2004). Therefore, given the unbinding nature of international laws and the impotence of global institutions (Krasner, 1985), they are responsible for the inability to ensure that the rich states will pursue altruistic policies in terms of distributive justice (WHO, n.d.-b). Therefore, communitarian and neorealist theorists may err at governments to ameliorate poverty within their respective states. In this pandemic time, it has become a moral duty of the developed nations to pursue the Cosmopolitanism approach to ensure global justice for all by waiving off the TRIPS.

### Indo-Africa: natural and strategic partnership

India–Africa health cooperation is multidimensional, comprehensive, and engages state, national, and sub-national actors to augment the pharmaceutical institutional capacities. Given the ongoing COVID-19, pandemic relief and equitable vaccine access efforts have emerged as the priority areas of collaboration, such as ensuring the export of low-cost generics, building health infrastructure, providing aid and technical assistance, to needy countries. Concomitantly, India and South Africa had the opportunity to help in navigating the complex patent regimes (WHO, n.d.-c).

India and South Africa have led third-world countries in raising their concerns in international forums since the end of colonialism. Given the prevailing COVID-19 critical scenario regarding the shortage of medicines and vaccines, third-world countries required the same leadership once again. India and South Africa have again emerged as the leaders of the third-world countries to meet the vaccine/medicine demand shortage. With the outbreak of COVID-19, the shortage of vaccines has become one of the major issues for not both countries, rather for the Global South. Moreover, these two countries have remained as the leaders of third-world countries. At this juncture, as a corollary of the preordained engagements, it became spontaneous for India and South Africa to show exemplary leadership concerning public health and have decided to raise at the global fora in WTO. The WTO Agreement on TRIPS is considered an instrument that brought about many drastic changes in the international standards relating to Intellectual Property Rights (IPR).

### Shortage of COVID-19 vaccines and medicines

COVID-19 has expanded worldwide, infecting more than 200 nations since the pandemic was proclaimed with 227,277,940 infected cases, 4,673,832 deaths, and 203,978,256 recovered cases of 16 September 2021 (WHO, 2020a). After the peak of the first pandemic wave, several countries have been entrapped in the second and third pandemic waves. The United States experienced three waves, that is, April and July 2020 and January 2021, the third more dreaded wave. The United Kingdom also passed through the three waves, in April, October to November 2020, and January 2021, respectively. Again, the third wave is the most critical one.

Currently, India is one of the countries that have been affected very seriously and critically, given the second wave of COVID-19. During the second wave of COVID-19, the pandemic has expanded tentacles every nook and corner of the country, approximately covering about 533 districts, covering almost 73% of the reporting cases. There are approximately 30 such districts of the Indian states such as Madhya Pradesh, Uttar Pradesh, Maharashtra, Tamil Nadu, and Bihar, whereas, however, 10–30 districts in 16 other states wherein each one of the districts has been reporting at least 10% to 20% positive cases as of August 2021. In this way, India has been accounting for nearly half of the world’s daily cases and becoming one of the places wherein one-fourth of the death of the world has taken place in India.

At the pandemic’s peak, it is clearly visible that there is an un-equitable distribution of vaccines between the developing and developed countries. The rich countries had hoarded the vaccines by signing the pre-production orders with the major pharmaceutical companies and even had already given shots to a considerable percentage of their population and turned the country into their normalized situation. However, developing and poor countries are constantly troubled by the lack of vaccines and other life-saving medical treatments and health care technologies. At the global level, efforts have been made to escalate the production of the COVID-19 vaccines globally. The WHO COVAX facility has adopted the motto, “No one is safe unless everyone is safe” (WHO, 2020b), to emphasize the need for equitable vaccination. Under the same facility, the goal was to supply the COVID-19 vaccines to about 100 low- to middle-income countries (LMICs), which could not afford the vaccines. It has also raised US$6.8 billion for purchasing and delivering the vaccines to participating countries congruently to the size of their populations (Mullard, 2020). The Facility also signed agreements with the vaccine manufacturers for supplying the 1.3 billion doses for 92 low-middle income countries during the first half of 2021 (So and Woo, 2020).

**Figure 1.**
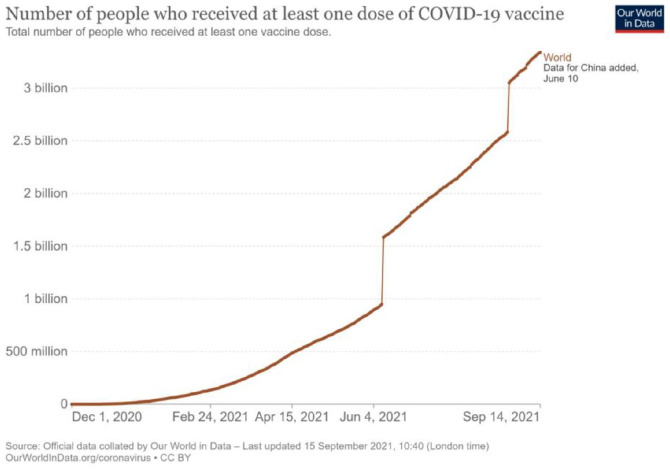
Graph showing the share of people globally who received at least one dose of COVID-19 vaccine (till 16 September 2021). *Source*: Our World in Data (accessed 2 September 2021). Retrieved from: https://ourworldindata.org/grapher/share-people-vaccinated-covid

**Figure 2.**
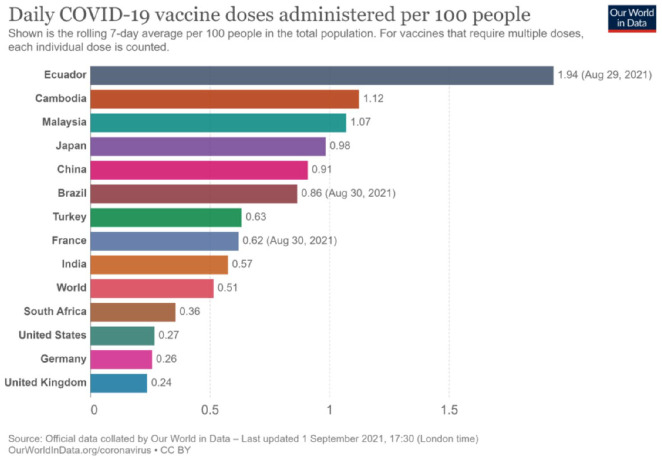
Graph showing countries with daily COVID-19 jabs administered for every 100 people (as of 2 September 2021). *Source*: Our World in Data (accessed 2 September 2021). Retrieved from: https://ourworldindata.org/grapher/daily-covid-vaccination-doses-per-capita

Despite these commitments, the pandemic has not yet been brought under control, and the situation has worsened due to a lack of vaccine affordability, accessibility, and equity among the LMICs. Of course, the internal milieu has been responsible for this situation; however, it was more exacerbated by applying the IPR. This argument is corroborated by the fact that some 16 countries that represent only 14% of the world’s population had gone on pre-order of more than 10 billion vaccine doses, or more precisely, one can say that about 51% of the available world supply by the mid-December 2020 (BBC, 2021). One more study (WHO, 2020a) had pointed out that notwithstanding having about 1% of the total global COVID-19 cases, countries like Australia, Canada, and Japan had collectively reserved more than 1 billion vaccine doses. The same study had raised concerns that the developed countries would be able to meet their vaccine demands in 2020–2021, whereas the poor countries may be deprived of the same until 2023–2024. As per one BBC Report (Wake, 2020), about 25% of the population in high-income countries (HICs) have already been vaccinated compared to only 0.2% in low-income countries (Figures 1 and 2). To make the situation more clear in this context, the stance of the COVAX Facility is sufficient as it has been committed to providing vaccines for more than 100 countries that had reserved only a few 100 million doses (Huneycutt et al., 2020).

Given the need for COVID-19 vaccines and its funding to vaccinate people in low-income countries during the first half of 2020, the WHO has partnered with the Coalition for Epidemic Preparedness Innovation (CEPI), the Bill and Melinda Gates Foundation, and Global Alliance for Vaccines and Immunizations (GAVI). These institutions had risen over US$20 billion in funds for the same (McCann and Gamio, 2021). The CEPI has also revised its equitable access policy concerning the funding. The major revisions have been put in place as the pricing of the vaccines may be set as low as possible for countries or territories that are or may be affected by pandemics for which CEPI funding was used to develop a vaccine. The vaccine manufacturing technologies must be shared with the CEPI, thereby assuming the responsibility for developing a vaccine if the company discontinued the same. The CEPI would have the IPR of the promising vaccines; share the financial benefits coming from CEPI-sponsored vaccine development, and re-invest to support its mission to provide a global public health benefit and data transparency (Loyo et al., 2021). Global Health Security Agenda, One Health, Universal Health Coverage, MDG, and SDG have been some of the major initiatives in the direction of equity of health.

Notwithstanding the several initiatives at the global level, about 67 countries, mostly falling from Africa and the Middle East regions, had not yet reported any vaccinations by mid-March 2021 (*The Times of India*, n.d.). Most of the poor and developing countries that rolled out the vaccination drives left the major chunk of the population without vaccination and only health workers or the elderly, have been prioritized. From Table 1, it is clearly evident that only 8% of the total population had the opportunity to get the first dose of the COVID-19 vaccine at the global level. The most glaring example of a shortage of vaccines is Venezuela (Human Right Watch, 2020) which has been collapsing, and at the current vaccination rate, it is likely to take 10 years.

### The Indo-South African patent diplomacy

The shortage of the COVID-19 vaccine is a major challenge in the present context. Poverty, lack of capital and Human Resources Development, poor health infrastructure, and TRIPS issues are major factors for the shortage or inaccessibility, affordability, and equity of the vaccines. The temporary waiver would facilitate the production of vaccines and cover up the shortage of the same vaccines, medicines, and so on, as argued by the proponents of the waive-off of the intellectual property (IP) proposal (WTO, 2016). The Agreement on TRIPS was brought into force on 1 January 1995. India and South Africa have already implemented their obligations under the TRIPS agreement.

The Indian Patent Act 1970 was amended in 1999, 2002, and 2005 to comply with the WTO TRIPS. Under the Act, the product patents extended in all technology areas such as food, medicines, chemicals, and microorganisms. However, the provisions such as exclusive marketing rights (EMR) have been made away. The new provision of the grant of compulsory licenses, pre-grant, and anti-post protests have been introduced (Communication from India and South Africa, n.d.). In the South African Patent Act 57 of 1978, the government has tasked the Companies and Intellectual Property Commission (CIPC) as the custodian of all new patent issues. The patent protection used to be granted for 20 years from the filing date of the non-provisional patent application as per the Patent Act 1978. Moreover, the TRIPS Agreement protocol has been intended to facilitate the developing and poorer WTO members to access affordable medicines, adopted in 2005. South Africa accepted the 2005 protocol amending the WTO’s Agreement on TRIPS on 23 February 2016 and became the fourth WTO member to do so in 2016 (Chandrasekharan et al., 2015).

It has been realized that the shortage of vaccines is one of the major reasons for the domestic landscape of inadequate health infrastructure and external factors for the continuity and inflated pandemic cases. Now, the question is how can the pandemic be controlled and combatted? The answer lies in the complete vaccination of people. However, the complete vaccination has not become possible given the various reasons and IPR as a barrier to the transfer of technology, production, distribution, and so on. To ensure health equity, affordability, accessibility, and availability, many national governments, regional bodies, and global health governance mechanisms have taken several initiatives. Some of them included WHO as the leader of the Global Incident Management Support Team (IMST) structure, the UN Crisis Management Team (UNCMT), and the founder of the Access to COVID-19 Tools Accelerator (ACT-A).

Notwithstanding several programs, GAVI has provided immediate funding to the health systems of many developing countries. Furthermore, the list is exhaustive, including multilateral bodies like the United Nations (UN), World Bank, International Monetary Fund (IMF), United Nations Children's Fund (UNICEF), the Group G-7, G-20, BRICS, QUAD, and individual nations pursuing through its global health diplomacy (GHD) negotiations. Despite these measures, ground realities have not changed much. Against this background, the relevance of India’s patent diplomacy holds ground.

In the joint proposal, India and South Africa had pointed out that the tentacle of the COVID-19 pandemic has been found widespread, affecting almost all WTO Members. As of 2 October 2020, “there is no vaccine or medicine to effectively prevent or treat COVID-19. All World Trade Organization, WTO (2020) Members are struggling to contain the spread of the pandemic and provide health care services. . . . . . .developing countries and least developed countries are especially disproportionately impacted (Usher, 2020). The study (DW, n.d.) found that IPRs, particularly patents, have been the major obstacles for developing country vaccine manufacturers to enter the vaccine market (Usher, 2020). During the pandemic, India and South Africa have been pursuing purposive health diplomacies, and proposing to waive off the IPs is one of many health diplomatic steps. India and Africa, the leaders of the third-world countries, members of the WTO, and compliant with the TRIPS, had submitted a patent waiver proposal in October 2020 for vaccines and medicines to the WTO’s Council for TRIPS. In this proposal, both countries demanded “a waiver from the implementation, application and enforcement” of several sections of the TRIPS Agreement about “prevention, containment or treatment of COVID-19.” Mustaqeem De Gama, the Counselor at the South African Permanent Mission to the WTO, has argued that “waiver proposal does it opens space for further collaboration, for the transfer of technology and for more producers to come in to ensure that we have scalability in a much shorter period of time.”

The proposal was introduced to the WTO’s TRIPS Council on 16 October 2020 and was re-discussed at a council meeting in November 2020. A study (Usher, 2020) pointed out that India and South Africa have called for the WTO members to temporarily suspend the IPR concerning COVID-19 medicines and vaccines. Through this waiver, the developing countries would be able to access and afford the COVID-19 vaccines and medicines and benefit from technology transfer. But very instantly, the reactions on the part of the rich countries came as very surprising. Showing insensitivity to the grim situation of the pandemic, the rich countries blocked (World Health Organization (WHO) & International Conference on Primary Health Care (1978: Alma Ata, USSR), 1988) the India–South Africa’s joint proposal for a temporary waiver on the IPR by arguing that it would stifle and discourage the innovations; research, and development of medicines and vaccines, which are urgently needed.

However, the same proponents argued that the rich countries would benefit more from the waive-off on IPRs techniques as they enter the pharmaceutical industry and markets. The poor and developing countries would become devastated by the pandemic. The SA Government had responded to such objections, pointing out how IP has created barriers to access the vaccine. The SA government quoted the legal battle between the Médecins Sans Frontières/Doctors without Borders (MSF) and Pfizer over its pneumococcal vaccine for treating pneumonia in India, whereby a patent has blocked manufacturing alternative versions of the vaccine. Similarly, Pfizer sued SK Chemicals Company of Bioscience in South Korea, which had developed the pneumococcal conjugate vaccine (PCV), forcing the Korean company to close production of PCV-13. Against these stances, the SA government argued that the same kind of situation would emerge with COVID-19 vaccines unless some concrete steps have not been taken to address the IP barriers (WHO, 1995).

### Global reactions

It is well known that WTO decisions are generally used to be taken through the consensus. More than 100 LMICs had already extended their whole-hearted support to the temporary waiver of support to the proposal. A group of 46 least developed countries responded positively to the joint proposal. The UNCTAD extended its support to the joint proposal call. The proposal was further strengthened and stimulated when about 350 civil society organizations or activists worldwide extended their support and asked WTO to positively take the proposal. However, there is still strong, stiff opposition on pharma giants, developed countries, including the European Union (EU). Some countries such as the United States and a few European nations that have opposed the proposal earlier have changed their stance and started supporting the India–South Africa joint waiver application at WTO. The major issue to be highlighted here is that the rich nations like the United Kingdom, the United States, Canada, Norway, and the European Union; major pharmaceutical multinational companies have outrightly rejected the proposal arguing that the IPRs are a must to foster and incentivize the new inventions of the vaccines, medicines, other life-saving technologies, and treatments.

However, these countries claimed and voiced that IPs could not be barriers to accessibility, affordability, and equity. They claimed that the accessibility and equity could be achieved by putting arrangements such as voluntary licensing, technology transfer arrangements, and the donor-funded COVAX Advance Market Commitment for vaccines. When we look at the more specific examples one by one, it is apparent that the European Union has not supported waiving the IPs. It is worth quoting the spokesperson of the European Union who argued that “There is no evidence that IP rights in any way hamper access to COVID-19-related medicines and technologies.” The United Kingdom has also taken the same stand and emphatically stated that “currently, the world direly needed accessibility to the new vaccines/medicines to control and combat the pandemic.” It further stated that “which is why a strong and robust multilateral IP system that can meet this challenge is vital.” Even the WHO has not taken a clear and crystal stand on the same.

Doctors without Borders—an international non-governmental organization (1971) working in 70 countries, has also been strongly advocating for the waiver of patents on COVID-19 medical tools and technologies from day 1, and the same was put in place. The MSF argues that the waive-off is justified given the two grounds: emergency health grounds and the inability of the LMICs to afford vaccines and treatments. Yuanqiong Hu (Senior Legal and Policy Adviser at the MSF Access Campaign) has argued that India–South Africa proposal would facilitate making necessary arrangements to produce medical equipment such as ventilators, masks, and protective gears. Even she had supported the need for technology transfers staunchly and mentioned that voluntary transfer through company-led initiatives had delivered limited results. AstraZeneca’s vaccine manufacturing agreements with Indian and Brazilian companies lack transparency about costs. She says that Pfizer and BioNTech, whose vaccine candidate has shown promising results, have shown no sign of licensing or technology transfer of their patented products. Pfizer told The Lancet that it would consider all viable options to ensure vaccines get to those who need them, but “a one-size-fits-all model disregards the specific circumstances of each situation, each product, and each country.”

The proponents of the patent waiver proposal realized and acknowledged that COVAX, funded by HICs, failed to provide timely and equitable access to COVID-19 vaccines. The same is corroborated by the fact that the COVAX has aimed to procure 2 billion doses of vaccine and share the same equally between HICs and LMICs. It has been found that the HICs have reserved 6 billion doses; meanwhile, the LICs, with a combined population of 1.7 billion people, have not yet signed a single bilateral vaccine deal. The same case goes with the ACT-A. The massive funding gap is haunting even this program, and thereby the set targets seem a distant daydream since only US$5 billion funds are available; meanwhile, $43 billion are still required for LMICs over the next year. De Gama has substantiated the above-cited stance by arguing that ACT-A has failed to provide the required supply of vaccines and not guarantee universal access as mandated by the Universal Health Coverage (UHC) and Global Health Security Agenda (GHSA).

## Discussion

### Patent diplomacy

Pandemic has created an ethical question for global justice. The lack of health availability of vaccines and medicines questions the functioning of the global health governance system. Several national, regional, and global governance mechanisms have been put in place to ensure Health for All. Mahler, the former Director-General (Director General, 1973–1983) of the WHO has highlighted the same, as healthcare equity is to be ensured or the services should reach all people in a given country. Moreover, Health for All stands to eliminate all the medical obstacles to health, such as the lack of doctors, hospital beds, drugs, and vaccines. He emphasized the continued progress in medical care; public health and Immunization must be for universal coverage (WHO, 2021). It was not just an ideal but also an organizing principle; everybody needs and is entitled to the highest possible standard of health. In this light, public health is one of the important global issues. When healthcare facilities, health equities are not available, then the question of global justice comes into play and becomes very important to contextualize the same, particularly in the context of the pandemic. Against the background of inaccessibility and non-affordability of vaccines, global justice has been relevant in the context of the ongoing pandemic and global health crisis.

What is the status of global justice, particularly in the context of the ongoing pandemic? The shortage of healthcare facilities in vaccines has created a moot question for global health justice. The IPR is considered as one of the barriers to health equity (Singh and Chattu, 2021). Therefore, the IPR suspension may provide a time being relief as a tool for health equity. Therefore, India and South Africa have floated an idea of waiving off IP rights to WTO, allowing generics and other manufacturers to produce vaccines and medicines until the citizens of the respective countries have not developed herd immunity. Beyond doubts, more than 100 countries, Nobel laureates, academicians, health rights activists, philanthropists (international, regional, and local), and human rights bodies had supported the joint proposal to waive the IPRs. However, at the same time, several European countries, including the United States, Australia, New Zealand, and Canada, have not come to terms or rather remained reluctant to vote for the same. Rather, they remained steadfast in favor of IPRs as well as their pharmaceutical gains (Chattu et al., 2021b). Moreover, The WTO Ministerial Conference (Doha Declaration) has also recognized the public health and private patents and further argued that the TRIPS agreement could not restrict the signatories from taking measures to protect public health. Notwithstanding all these frameworks in place, the ground realities in the context of the pandemic have been defying public health dreams, particularly in health equity.

### Global justice

Global justice is an element of normative global health governance dynamics that focuses on all the moral obligations from the world’s rich to the poor. Thus, global justice stands to redistribute wealth to the poor people to eliminate global poverty and other socio-economic concerns. The US Health and Human Services Secretary Xavier Becerra (while participating in WHA 74) said Universal Health Coverage is “a right and not a privilege.” Even public health has been prioritized and institutionalized, considering global health justice. Several programs, policy initiatives, campaigns have been launched, such as health for all and health equity. The International Covenant on Economic, Social, and Cultural Rights (Article 12) had acknowledged, “The right to the highest standard of physical and mental health.” It gives some directives for states to prevent, treat, and control epidemics, endemics, occupational, and other diseases. The Universal Declaration of Human Rights (Article 25) conceived health as a human right and postulated to achieve “a standard of living adequate for the health and well-being of himself and of his family.” The TRIPS agreement also underlined (Article 7) that the exercise of intellectual property rights should be carried out in a “manner conducive to social and economic welfare, and a balance of rights and obligations.” Furthermore, TRIPS Article 8.1 emphasized that the, “Members may, in formulating or amending their laws and regulations, adopt measures necessary to protect public health and nutrition” keeping these measures are in tandem with the TRIPS agreement (WTO, n.d). The WHO says, “the health is one of the fundamental rights of every human being . . . The extension to all peoples of the benefits of medical, psychological and related knowledge is essential to the fullest attainment of health” (Constitution of the WHO) (WHO, 1995).

The above discussion can be summarized as supporting the right to achieve the highest standard of physical and mental health as defined by the WHO. Moreover, to prevent, treat, and control pandemics or diseases for health security and to support or advocate health as a human right, we have to exercise the IPRs in a conducive way to support the social and economic welfare of all the global citizens (Chattu et al., 2021a). Hence, there is an urgent need to adopt necessary measures to protect public health, as health is one of the fundamental rights of every human being, and look through the prism of global justice.

The IPR is considered one of the major barriers to health equity. For example, more than 11 billion COVID-19 vaccine doses are needed to administer two doses to 70% of the total population. It is reported that until now, more than 8.6 billion orders have been placed (United Nations, 2021). However, the important point that has to be highlighted is that out of these 8.6 billion vaccines, 6 billion will go to the rich countries, and 2 billion only go to the developing countries wherein 80% of the global population have been living. Meanwhile, the developed countries, having only 13% of the total population, had procured more than half of the future supply of the COVID-19 vaccine contenders, which is far more than more they require (Oxfam International, 2020). Canada has enough COVID-19 vaccine doses to cover each citizen five times over, while the fate of 67 poor countries remains undecided. It is further substantiated by taking the example of Canada, which has reserved five times over, while the developing countries have not been able to vaccinate even with a single dose. These huge gaps in access have necessitated the waive-off of the IPRs. Besides, the WHO Chief has portrayed the ground reality as the “World Is Far Behind” in the context of reaching the level of UHC (WHO, n.d.-d).

Against this backdrop of bad to a worse situation of health equity, India and South Africa being the leaders of the third world, realized the gravity of the situation and had put up the joint proposal (IP/C/W/669) to waive off IPRs temporarily and enable to expedite the development of vaccines, medicines, and diagnostics for containment, prevention, and treatment of COVID-19 (Chattu et al., 2021a, 2021b; Singh and Chattu, 2021; WHO, n.d.-a). Moreover, the proposal covers all the medical products direly needed for testing, treating, or preventing COVID-19 must be included under such a waiver (McCann and Gamio, 2021).

India and South Africa have been using diplomatic channels either bilaterally, regionally, or globally to get through the proposal. Moreover, both countries have been using public health as part of the soft diplomacy of their foreign policy at the individual level. India has provided COVID vaccines to more than 100 countries through its “Vaccine Maitri” diplomacy (Mol et al., 2021). The patent diplomacy gradually began to bear fruit once it was supported by more than 120 countries, Nobel laureates, academicians, over 350 civil society organizations, activists, and international organizations such as Joint United Nations Programme on HIV/AIDS (UNAIDS), Unitaid (A global health Initiative founded in 2006), and MSF (*The Times of India*, n.d.). UNCTAD also supported the proposal and even urged WTO member countries to do the same. Congruently, the WHO wholeheartedly lent its support to the proposal and by the WHO Chief saying that “To ease international & intellectual property agreements . . . available to all who need them at an affordable cost.” India and South Africa take the lead further by arguing before the TRIPS Council that “Given the current global emergency, WTO Members must work together . . . distribution of medical products essential to combat COVID-19” (WHO, n.d.-a). Therefore, if the TRIPS waiver proposal is approved, the access to essential COVID-19 medicines, technologies, and diagnostics will improve drastically (Chattu et al., 2021b; Loyo et al., 2021; Singh and Chattu, 2021; Taghizade et al., 2021). Chattu et al. have highlighted that though it is easy to talk about inequalities and inequities and incorporate them into policies, the COVID-19 pandemic provides an opportunity for the world to demonstrate its solidarity for “Health for All” (Javed and Chattu, 2020).

## Conclusion

From the above discussion, it is clear that the IPR is one of the major bottlenecks for health equity. India and South Africa have been making wholehearted efforts to garner diplomatic support for the TRIPS waiver. However, considerable states and non-state actors have been supporting the proposal, still miles to go ahead to convince the developed and pharmaceutical giants to extend their support for waiving off the IPRs. Gradually, these countries would or could realize that India and South Africa’s joint proposal is a major step in the direction of health equity, health for all, one health, UHC, health as a human right, reducing the out-of-pocket expenditure, and protecting humanity at large. As a result, the proposal has the potential to catalyze health equity and aid in the pursuit of global justice.

The acceptance of the waiver proposal would also result in mass production and fair distribution, making it available to everyone regardless of their status. It would also enable the developing countries to produce additional billions of doses in the minimum period, ensuring timely availability and accessibility everywhere across the globe. In addition, profit can be earned later on the generation sustains; therefore, the developed countries, international and regional organizations, and major pharmaceutical companies must support the joint proposal to protect public health. Monopolies on vaccines must be done away with sharing “know-how” with the developing countries for mass production and reducing the prices. Global justice will prevail once there is a fair distribution of vaccines and essentials. However, it is not impossible because, for the most part, states have provided public funds to pharmaceutical companies for research and development (R&D). The paper concludes by supporting the ideas of former Indian Prime Minister late Mrs Indira Gandhi while speaking at the World Health Assembly, where she rightly argued that “My idea of a better-ordered world is one in which medical discoveries would be free of patents, and there would be no profiteering from life or death.” Nations should strive to find solutions to ensure equitable access to the COVID-19 related drugs, medical supplies and vaccines while allowing for profit-making by the pharmaceutical industry.
